# The impact of multi-person virtual reality competitive learning on anatomy education: a randomized controlled study

**DOI:** 10.1186/s12909-020-02155-9

**Published:** 2020-10-05

**Authors:** Yi-Chun Du, Shih-Chen Fan, Li-Cheng Yang

**Affiliations:** 1 Department of Biomedical Engineering, National Cheng Kung University, No. 1, University Rd., Tainan, 70105 Taiwan; 2 Medical Device Innovation Center, National Cheng Kung University, No. 1, University Rd., Tainan, 70105 Taiwan; 3 Department of Occupational Therapy, College of Medicine, I-Shou University, No.8, E-Da Rd., Kaohsiung, 82445 Taiwan; 4 Department of Electrical Engineering, Southern Taiwan University of Science and Technology, No. 1, Nantai St., Yungkang Dist. Tainan, 71005 Taiwan

**Keywords:** Virtual reality (VR), Anatomy, Medical education, Competition, Situated learning

## Abstract

**Background:**

Anatomy is one of the core subjects in medical education. Students spend considerable time and effort on learning the requisite anatomy knowledge. This study explored the effect of a multiple-player virtual reality (VR) gaming system on anatomy learning.

**Methods:**

18 participants were randomly assigned into 3 learning conditions: (1) a textbook reading control group (CG), (2) a single-player VR (SP) group; and (3) a multiple-player VR (MP) group. The participants studied anatomy for 5 days, and completed a multiple-choice test on Days 1, 5, and 12. In the VR environment, the participants used handheld controllers to move the simulated tissues. The mission of the game was to complete puzzles of a human body. The SP and MP groups filled out a motivation inventory on Day 5. The scores on the multiple-choice test, the correct assembly rates, and the motivation inventory scores were analyzed using the 2-way ANOVA or independent *t*-test to compare group differences.

**Results:**

There was a significant interaction effect of group and timepoint (*p* = 0.003) in the multiple-choice test. In the CG, the scores on Day 1, Day 5, and Day 12 were significantly different (*p* < 0.001). The scores on Day 5 were significantly higher than those on Day 1 (*p* < 0.001). Although the scores declined slightly on Day 12, they were still significantly higher than those on Day 1 (*p* < 0.001). The SP and MP groups had similar results (*p* < 0.001, *p* < 0.001). The differences between the groups were only significant on Day 12 (*p* = 0.003), not Day 5 (*p* = 0.06). On Day 12, the scores of the MP group were higher than those of the CG (*p* = 0.002). The SP group and MP group had high scores on the interest, competence, and importance subscales of the motivation inventory. Both VR groups considered the system to be fun and beneficial to their learning. However, the MP group reported higher stress levels than the SP group.

**Conclusion:**

The results indicated that the proposed VR learning system had a positive impact on the anatomy learning. Although the between-player competition caused higher stress levels for the VR groups, the stress could have been a mediator of their learning outcomes.

**Trial registration:**

ETRD, ETRD-D-19-00573. Registered 20 December 2018, http://www.edah.org.tw/irb/index.htm

## Background

Learning in authentic environments is key to successful learning. As Brown, Collins and Duguid have stated, “knowledge is situated, being in part a product of the activity, context, and culture in which it is developed and used” [1]. This notion is of essential importance in medical education. Medical education relies heavily on the contexts in which instruction occurs, including the associated interpersonal interactions and artifacts [2]. Virtual reality (VR) systems have been applied to medical education in many subjects, such as anatomy, orthopedic surgery, dental practice, emergency medicine, etc. [3]. Anatomy is one of the fundamental issues in medical education. Medical students are required to learn the anatomy of the human body by heart, so that as professionals, they can differentiate abnormal structures from healthy tissue. Medical students must memorize the relevant terms, shapes, appearances, locations, and sizes of the human organs. The terms used in anatomy originate from ancient Latin and Greek. Students spend considerable time and effort on learning the requisite anatomy knowledge.

Several research teams used VR for anatomy instruction. In one study, students who used a 3D anatomical ear model scored significantly higher on a quiz about the relationships within the ears [4]. In another study, the direct manipulation of VR facilitated the embodiment of knowledge of anatomy structures compared with passive viewing only [5]. Still other research found that augmented reality with tangible manipulation of the human skull helped students in memorizing the skull’s structure [6]. In short, VR environments can improve learning efficacy and motivation [7]. With the guidance provided by computational images or movies, students can undergo fully immersive situational experiences [8]. However, only a limited amount of studies considered the effects of the competitive element of VR programs on medical education. For example, a single-player VR program may have a lack of interactions, which could, in turn, cause users to feel bored.

In contrast, competition among players might increase the enjoyment and attractiveness of learning tasks [9]. In other words, competitive elements could be sources of motivation [10]. The purpose of the current study was to investigate the use of competitive learning in anatomy instruction to assess the effectiveness of a VR system employing such learning on students’ motivation levels.

### Theoretical framework for “competitive learning”

According to Deutsch’s theory, classroom learning frequently involves two types of group processes: cooperative and competitive operations [11]. In a competitive group, the actions of students are negatively aroused, and students tend to obstruct one another to win. Cheating, callousness, and selfish behaviors might thus be added to the process [12]. Therefore, some researchers see “competition” as something that undermines intrinsic motivation [13]. Relatedly, the self-determination theory pointed out that intrinsic motivation was the key to successful learning [14]. Competing to win a trophy entails motivation based on a source of external loci of causality [15]. Therefore, it was believed that competition could hinder intrinsic motivation [12]. However, later studies of the self-determination theory proposed that competition can either be beneficial or harmful to intrinsic motivation, depending on the interpersonal interactions and individual characteristics of those involved in the competition [16, 17]. If the interpersonal interactions of the competition are not stressful, but rather, allow autonomy, then the competition can be internalized more towards the intrinsic end than the external end on the motivation continuum. A chance of victory might increase the sense of competence and promote intrinsic motivation in those involved in the competition, especially for students with an achievement orientation [17]. However, when the requirements of a game or learning task are too advanced for learners’ skills or resources, the learners will feel stress [18]. Taken together, the research above has indicated that learning performance is related to the stress levels that students experience. To date, relatedly, the debate regarding the effects of competition has not been conclusive. The current study thus sought to explore the impact of competition on medical learning in the context of a VR system.

### The current study

The current study investigated the effects of three different learning conditions on the learning of anatomy. The first purpose of the study was to determine the difference in the effects of textbook reading and VR learning. The second purpose was to explore the differences between single-player and multiple-player VR learning. Our first hypothesis was that the VR learning would have better effects on learning than the textbook reading. Our second hypothesis was that the multi-player VR condition would increase learning to a higher degree than the single-player VR condition. Our research question was, would the multiple-player VR condition stimulate better results than the conventional or single-player VR conditions?

## Methods

### Participants

**Fig. 1:**
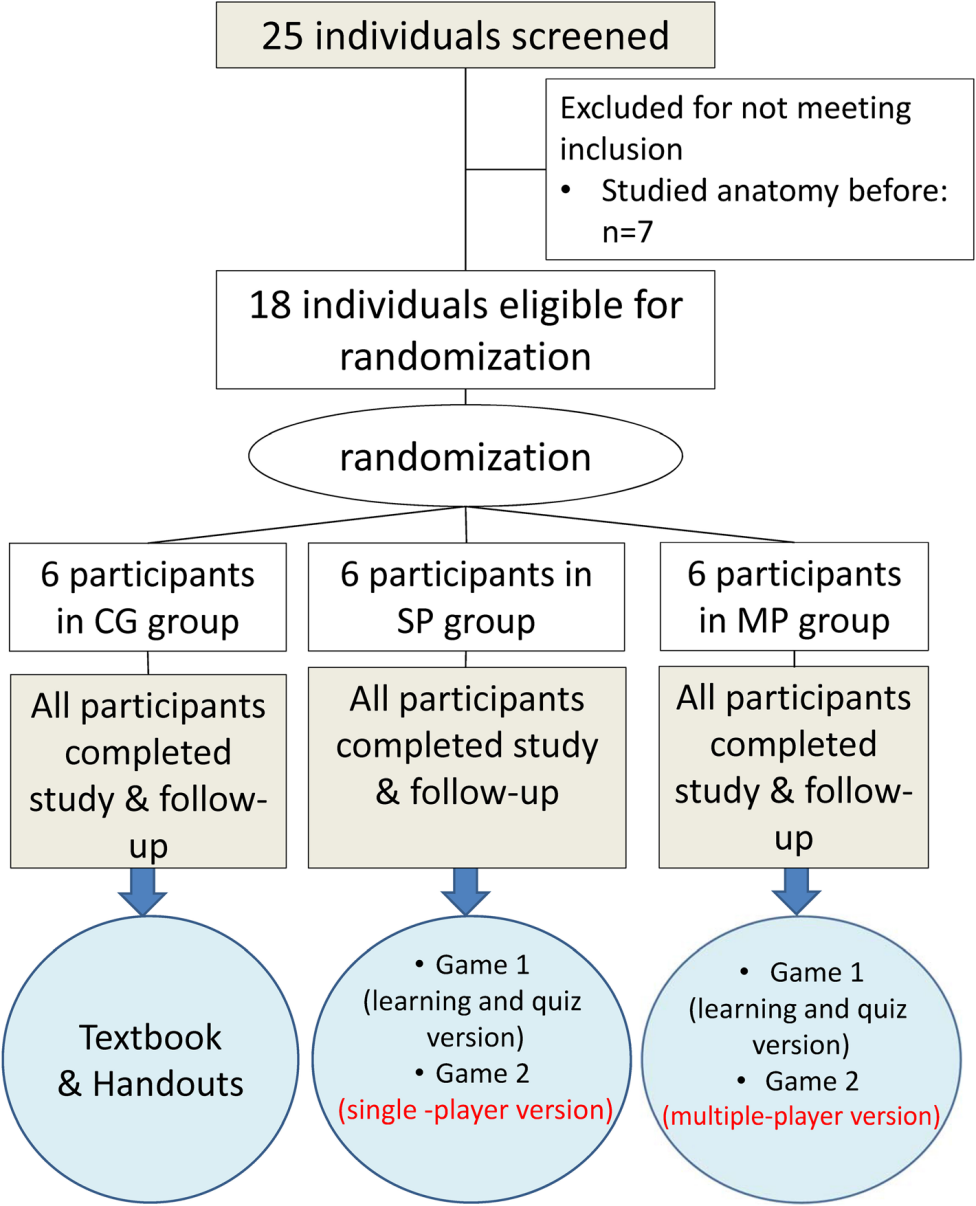
Flow chart of the study protocol

The participants were recruited through advertisements posted around the I-Shou University and Southern Taiwan University campuses. Participants who had no previous anatomy knowledge were eligible. 25 university students expressed their willingness to participate in the study. However, 7 students were excluded from the study because they had learned anatomy before. Figure 1 shows the flow of participants through the trial. 18 students met the criteria and enrolled in the study. The participants were randomly assigned into one of three groups: the control group (CG), the single-player VR (SP) group, or the multi-player VR (MP) group. There were 6 participants in each group. A list of computer-generated random numbers was generated using a simple random number generator. All the participants were blinded to the experimental hypothesis during the experiment. All 18 participants completed the five-day study period and the follow-up tests. The response rate was 100%. The 18 participating college students included 14 male students (77.8%) and 4 (22.2%) female students. All the participants’ had similar levels of prior experience in playing computer or smartphone games. 10 of the 18 students (55%) were from the college of engineering, and 8 (45%) were from the college of medicine. The 8 students from the college of medicine were from either the Department of Health Management or the Department of Biological Science. The courses of those two departments did not include anatomy courses, even though the two departments were part of the college of medicine. The mean age of the participants was 21.93 years, with a standard deviation of 0.97 years. Written informed consent approved by the Eda Hospital ethics committee was obtained from each participant prior to his or her inclusion in the study.

### The 3D modeling and animation

**Fig. 2:**
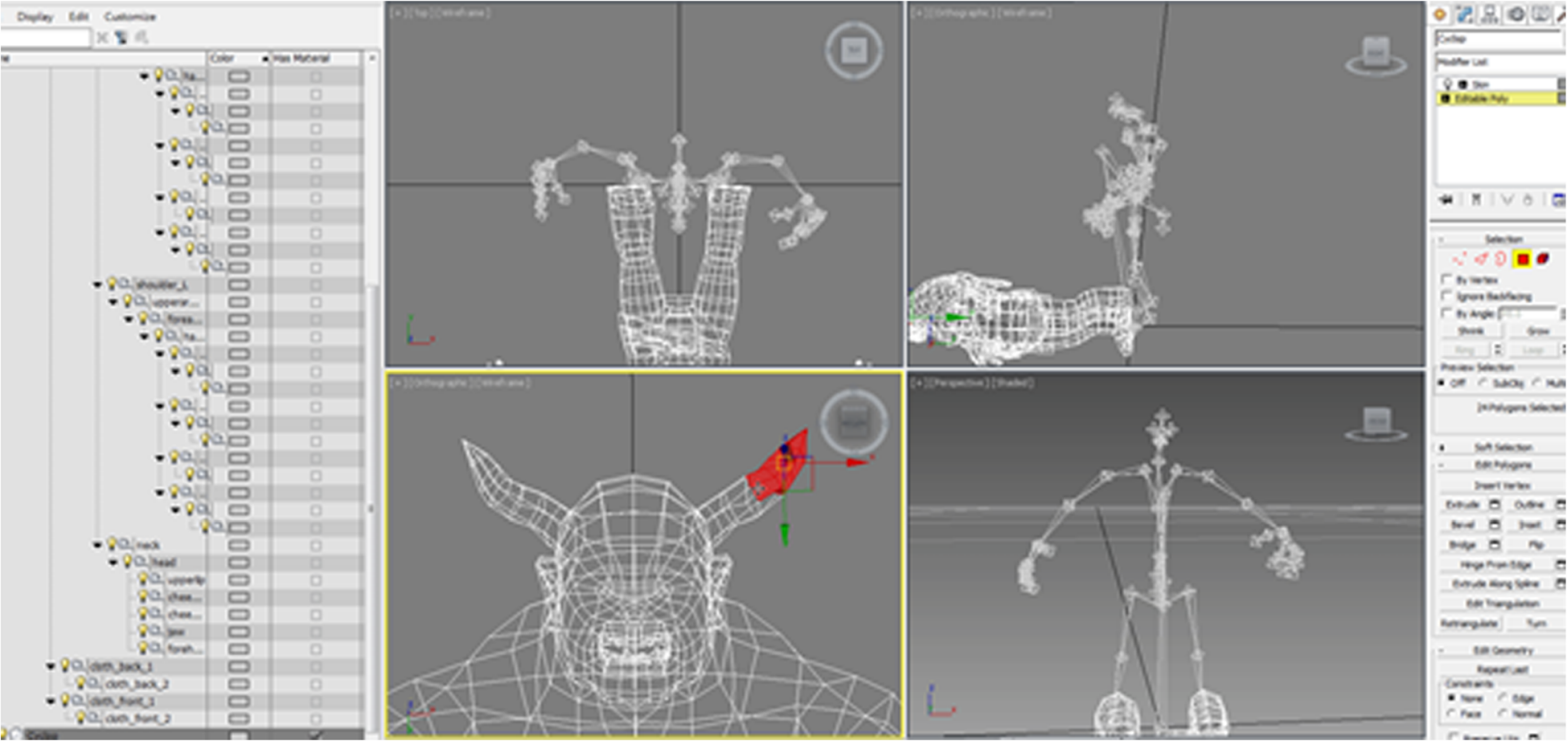
The 3D modeling in the system

The virtual reality gaming system was based on the HTC Vive system (HTC Corporation, Taiwan). The Vive consists of two controllers, a head-mounted display, and two infrared laser emitter units. The 3D models of bones and muscles in the system (Fig. 2) were made with the Autodesk 3DS Max software (Autodesk Media and Entertainment, New York). A total of 25 bone and 25 muscle models of the extremities and the trunk were built. We used Unity (Unity Technologies, San Francisco), a cross-platform video game engine, to record the animation of the 3D molding. The Unity engine helped us in assembling the system assets into scenes, audio cues, special effects, lighting, and animation, while allowing for simultaneous play.

### The content of the game

**Fig. 3:**
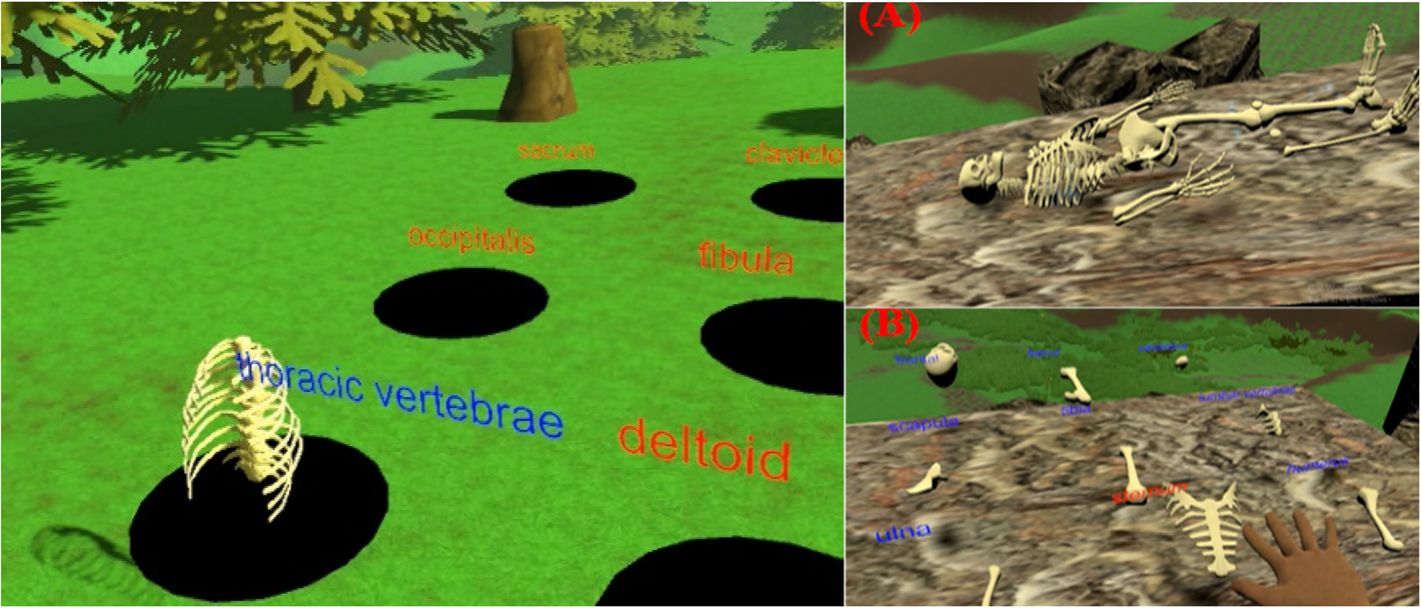
Screenshot of the game

There were two games in the system. Game 1 helped the participants to memorize the names of muscles and bones. The participants observed 3D models of bones and tissues using the VR headset. The participants used the touchpad on the controllers to rotate the anatomy images and used the grip button to zoom in and out. The screen displayed 9 holes with the names of different bones on top of the holes. The participants pressed the trigger on the controllers to picked up the right bones and released the trigger to throw them into the holes (Fig. 3). The participants could play the game with or without visual and auditory cues regarding correct answers. Both the SP and MP groups participated in Game 1. There were no differences for the participation of SP and MP groups in Game 1.

Every session of the game contained 5 rounds of play, resulting in a total of 45 questions in a session. The correct assembly rate was calculated as “Accuracy = ((45-WrongAssembly)/45) × 100%.

Game 2 helped the participants to learn the correct positions of muscles and bones. The participants assembled the puzzles of bones and flesh into a complete human body. If one puzzle was organized in the proper arrangement, the participants could send out one guard to protect the castle. Meanwhile, the computer would send out an enemy to attack the castle. The longer the participants could sustain and defend the castle, the higher the scores the participants received.

Game 2 had two versions: a single-player version and a multiple-player version. The SP group only played the single-player version, with the given participant fighting against the computer. In contrast, the MP group only played in multiple-player version, with the given participant fighting against several real players online.

### Experimental procedure

The experimental procedure included two phases. In the first phase, each group had 5 days to study. The control group studied the textbook “Atlas of Anatomy” [19] and a 5-page handout for 30 min each day. The handout specified the name of the bones and muscles in the test scope. The test scope included the names, positions, and pictures of the same 25 bones and 25 muscles that were included in the VR game. The VR groups played the game for 30 min each day. The VR game system recorded the correct assembly rate every day. In the second phase, the retention phase, all three groups stopped studying for 7 days after completing the 5 days mentioned above. Knowledge-based multiple-choice tests on anatomy were administered on Day 1, Day 5, and Day 12 to all three groups. The sample question in the multiple-choice test was “Name the muscle highlighted in red.” There was a picture of upper extremity muscles to one side of the question. The deltoid muscle was colored in red. The participant chose the correct answer from the list accordingly. The participants in the VR groups (that is, the SP and MP groups) also filled out a motivation inventory on Day 5 only as an evaluation of their motivation to learn. The motivation inventory was adapted from the Intrinsic Motivation Inventory [20]. The inventory contained 4 subtests: the interest, competence, importance, and stress subtests. There were 22 questions in the inventory. The response choices for each question ranged from seven (strongly agree) to one (strongly disagree). The total possible scores on the enjoyment, competence, importance, and stress subscales were 42, 42, 35, and 35, respectively.

### Data and analysis

The demographic data of the participants were compared using descriptive analysis. We first ran a normality test on the outcome measures (that is, the scores on the multiple-choice test, the correct assembly rates, and the scores on the motivation inventory). The distribution of scores for the multiple-choice test was normally distributed. We then used a 3 (group) × 3 (timepoint) between-within subject analysis of variance (ANOVA) to analyze the scores on the multiple-choice test. The significance level α was set at 0.05. If there was an interaction effect between group and timepoint, the simple main effect of the two independent variables would be analyzed with an adjusted α at 0.017. Bonferroni’s pairwise comparison was used to determine the differences between the groups. Although the correct assembly rates did not have a normal distribution, we still used a 3 (group) × 5 (timepoint) ANOVA to analyze the correct assembly rates. Using a two-way ANOVA would simultaneously produce effects estimates and significances for within-person change over time, and that is an appropriate choice as compared to a nonparametric test. The data of the motivation inventory were distributed normally, except for the stress subtest data. To include the analysis results of the all the subtests in the same table, we used the independent *t*-test to analyze the scores of the motivation inventory.

## Results

### Multiple-choice test results

Table 1 lists the scores of the 3 groups on the multiple-choice test. A significant interaction effect between group and timepoint was found (*p* = 0.003) (Table 2). In the CG, the scores on Day 1, Day 5, and Day 12 were significantly different (*p* < 0.001). The scores on Day 5 were significantly higher than those on Day 1 (*p* < 0.001). Although there was a small decline for the scores on Day 12, the scores on Day 12 were still significantly higher than those on Day 1 (*p* < 0.001). The results described above were also found for the SP and MP groups (*p* < 0.001, *p* < 0.001). On Day 1, the scores of the 3 groups were not significantly different (*p* = 0.911). Otherwise, the differences between the groups were only significant on Day 12 (*p* = 0.003), not Day 5 (*p* = 0.06). On Day 12, the scores of the MP group were higher than those of the CG (*p* = 0.002). These results revealed that the effect of VR learning was not significant on Day 5, but was stronger on Day 12.

**Table 1:** Correct assembly rates and scores for the multiple-choice test and the motivation inventory

	Day 1				Day 5						Day 12				
	Mean	SD		95% CI	Mean		SD		95% CI		Mean	*SD*		95% CI	
Multiple-choice Test															
Control Group	40.5	4.8		*36.6 ~ 44.4*	75.2		3.2		*72.2 ~ 77.7*		62.5	4.8		58.7 ~ 66.3	
Single Player	39.5	4.1		*36.2 ~ 42.8*	79.2		5.9		*74.4 ~ 83.9*		70.0	4.4		66.5 ~ 73.5	
Multiple Player	41.0	8.4		*34.2 ~ 47.8*	83.2		6.3		*78.1 ~ 88.2*		75.8	6.9		70.5 ~ 81.3	
	Day 1			Day 2			Day 3			Day 4			Day 5		
	Mean	SD	95% CI	Mean	SD	95% CI	Mean	SD	95% CI	Mean	SD	95% CI	Mean	SD	95% CI
Correct Assembly Rates															
Single Player	5%	9%	3% ~ 23%	25%	26%	4% ~ 77%	49%	37%	20% ~ 122%	63%	33%	37% ~ 127%	81%	14%	70% ~ 110%
Multiple Player	16%	24%	4% ~ 63%	64%	27%	42% ~ 116%	88%	12%	78% ~ 112%	93%	5%	89% ~ 103%	95%	7%	89% ~ 109%
	Enjoyment			Competence				Importance			Stress				
	Mean	SD	95% CI	Mean	SD	95% CI		Mean	SD	95% CI	Mean		SD	95% CI	
Motivation Inventory															
Single Player	35.0	3.2	32.5 ~ 37.5	33.5	2.3	31.4 ~ 36.2		30.5	2.7	30.8 ~ 36.5	7.0		2.3	5.2 ~ 8.8	
Multiple Player	37.7	1.6	36.4 ~ 39.0	33.7	3.6	30.8 ~ 36.5		32.0	2.0	30.4 ~ 33.6	19.5		4.8	15.6 ~ 23.4	
*p* value	0.096			0.926				0.304			0.000*				

*Abbreviations*: *SD* Standard Deviation, *CI* Confidence interval

*p* < 0.05

**Table 2:** The *two-way ANOVA* results of the multiple-choice test and the *t-test* results of the correct assembly rate

Two-way ANOVA	*SS*	*df*	*MS*	*F*	*p*		
**The multiple-choice test**							
time	14,700.0	1.4	10,294.7	725.8	0.000		
time*group	258.2	2.9	90.4	6.4	0.003*		
Error	303.8	21.5	14.2				
group	476.9	2.0	238.5	3.2	0.070		
Error	1122.1	15.0	74.8				
Simple main effect	*SS*	*df*	*MS*	*F*	*p*	*Group Comparison*	*Post-hoc p-value*
Day 1	7.0	2	3.5	.094	.911		
Day 5	192.0	2	96.0	3.408	.060		
Day 12	536.1	2	268.1	9.009	**.003**^**+**^	CG vs. SP CG vs. MP SP vs. MP	.075 **.002**^**+**^ .187
CG	3692.4	2	1846.2	148.6	**.000**^**+**^	Day 1 vs. Day 5 Day 1 vs. Day 12 Day 5 vs. Day 12	**.000**^**+**^ **.001**^**+**^ **.000**^**+**^
SP	5175.4	2	2587.7	303.6	**.000**^**+**^	Day 1 vs. Day 5 Day 1 vs. Day 12 Day 5 vs. Day 12	**.000**^**+**^ **.000**^**+**^ **.006**^**+**^
MP	6090.3	1.1	5567.3	322.8	**.000**^**+**^	Day 1 vs. Day 5 Day 1 vs. Day 12 Day 5 vs. Day 12	**.000**^**+**^ **.000**^**+**^ **.000**^**+**^
Two-way ANOVA	*SS*	*df*	*MS*	*F*	*p*	*Group Comparison*	*Post-hoc p-value*
**The correct assembly rates**							
Group	1.1	1	1.0	6.3	0.031*	MP > SP	
Error	1.6	10	0.2				
Timepoint	4.7	1.9	2.4	55.6	0.000*	Day 1 vs. Day 2 Day 1 vs. Day 3 Day 1 vs. Day 4 Day 1 vs. Day 5 Day 2 vs. Day 3 Day 2 vs. Day 4 Day 2 vs. Day 5 Day 3 vs. Day 4 Day 3 vs. Day 5 Day 4 vs. Day 5	**0.002**^**+**^ **0.000**^**+**^ **0.000**^**+**^ **0.000**^**+**^ **0.016**^**+**^ **0.004**^**+**^ **0.000**^**+**^ **0.012**^**+**^ 0.026 0.256
Timepoint* Group	0.3	1.9	0.1	2.6	0.102		
Error	0.9	19.3	0.04				

*Abbreviations*: *SS* sum of square, *df* degree of freedom, *MS* mean square, *CG* control group, *SP* single-player group, *MP* multiple-player group

Note. *α* = 0.05 at the two-way ANOVA test

*α* = 0.017 at the simple main effect test

*p < 0.05

^+^*p* < 0.017

### Correct assembly rates in the VR game

**Fig. 4:**
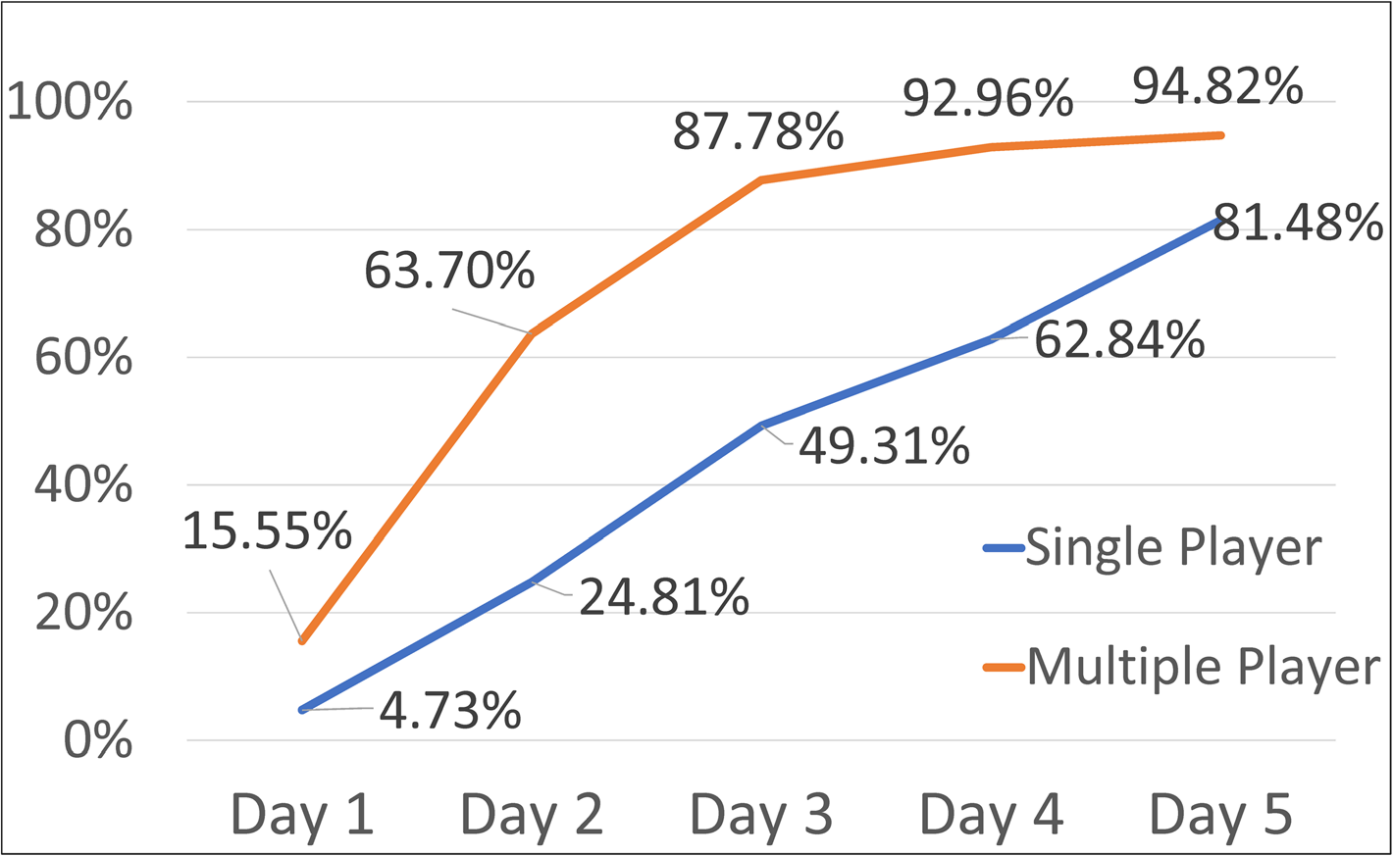
Correct assembly rates of the two VR groups

The correct assembly rates on Day 1 to Day 5 were shown in Fig. 4. Descriptive statistics of the correct assembly rates are listed in Table 1. Table 2 shows the 2-way ANOVA results for the correct assembly rates. There was no interaction effect between the variables of group and timepoint (*p* = 0.102). The main effect of group was significant (*p* = 0.031). The MP group had significantly higher correct assembly rates than the SP group. The main effect of timepoint was also significant (*p* < 0.001). The post-hoc test of timepoint revealed that there were significant differences among Day 1, Day 2, and Day 3 (*p* = 0.002, *p* < 0.000, *p* < 0.000). However, the discrepancies among Day 3, Day 4, and Day 5 were not significant (*p* = 0.026, *p* = 0.256).

### Intrinsic motivation inventory results for enjoyment, competence, importance, and stress

The two VR groups both had high scores on the motivation inventory, except for the stress subscale (Table 1). There was no significant difference between the SP and MP groups on the enjoyment, competence, and importance, subscales (*p =* 0.096, *p =* 0.926, *p =* 0.304). However, the MP group had a significantly higher level of stress than the SP group (*p <* 0.001).

## Discussion

The first hypothesis that the VR groups would have better learning performance than the CG was supported. For the multiple-choice test, the scores of the VR groups increased after using the system, although their progress was not shown immediately on Day 5. However, the MP group had significantly higher scores than the CG on Day 12. Therefore, the VR groups had better memory retention than the CG. The students in the VR groups could successfully embody virtual representations of anatomical structures with active manipulation. The 3D virtual system was thus an excellent tool for developing the learners’ spatial awareness [21, 5]. In our study, the VR group participants used handheld controllers to move and rotate the models of bones and muscles, which provided a natural form of manipulation. Our VR environment strengthened the spatial-related knowledge of the human body among the VR group students. With a VR system, students can repeatedly engage in anatomy learning before touching real cadavers. Cadaver resources are limited and expensive. In contrast, our VR system provides practice opportunities without time and geographic constraints. In the future, we could determine the correlation between the correct assembly rate and the score on the multiple-choice test. Relatedly, teachers could use our system to assess the performances of students remotely, and teachers could predict students’ performances based on their correct assembly rates.

The second hypothesis of the present study was that competition could increase learning. Our results also supported this second hypothesis. The correct assembly rates showed that the MP group had a higher correct assembly rate, on average, than the SP group, especially during the first 3 days of playing. A previous study found that with competition, the usage rate of a VR simulation system for surgical residents was increased by 23% [22]. On the other hand, when competition imposed a “must-win” climate on students using a VR laparoscopic training program [23], the motivation of the students to use the program was lowered because the students easily perceived the competition to be more controlling than informational in nature. In our study, in contrast, the competition was presented in a gaming climate. The students in our study perceived the competition to be playful and fun. The students often chatted and discussed how to win against each other, and as a result, our system established a learning community for the students. As such, the competition stimulated their learning potential. The Latin root for competition means “to seek together.” Therefore, during the process, the learners shared and interacted with their opponents and played the game in question together [24]. This could be the reason that competition was beneficial to learning in our study. In the future, the competition can be made more complex and transformed to consist of competition among teams, for example, 3 × 3 competitions. In that case, students could receive the benefits of learning through cooperation and competition at the same time. Moreover, the use of structured feedback, such as a checklist with details about the target knowledge or technique, could be further added in the learning process [25].

Some researchers have found that the competition element may cause different effects on learners of different genders. One study found that males experienced positive emotional responses during competitive rather than cooperative play, whereas females had no difference in their responses to competitive or cooperative games [26]. In our study, most of the participants were male, with only 22.2% of our participants being female. This factor constituted a limitation of our research. Therefore, in future research, more female participants should be included.

In the present study, the competition resulted in more stress being placed on some of the participants, and this increased stress had a positive effect on their learning. Reeve and Deci believed that self-determination is rooted in inner motivation, such that stress from competition could diminish internal motivation and, as a result, decrease self-determination and learning effects. However, the category, nature, direction, and amount of the stress experienced by students have different impacts on their learning results. Role ambiguity, role conflict, and hassles have been found to be related to the adverse effects of stress on learning [27]. Hindrances and challenging stressors have likewise been found to impact students in different ways [16]. Such stressors can be either harmful or beneficial, as learners seek to increase their levels of attention and speed up their information processing to evaluate and manage the given situation effectively. In our study, the performances of the participants in both VR groups were higher than the overall average scores, even though the stress levels were higher in the competitive group than in the SP group. Therefore, we concluded that while the competition in our study may have caused stress, the stress stimulated the inner motivation and learning of those who experienced it.

The limitations of our study included the small sample size, which might have diminished the statistical power of our research. Therefore, future research should be conducted with a larger sample size. At the same time, the 18 participants in our study were either from a college of engineering or a college of medicine. In the future, participants with various backgrounds should thus be included to increase the generalizability of the results. As cross-disciplinary learning has become one of the more prominent learning styles, a future study could seek to validate the effectiveness of our VR game for students of various disciplines. Furthermore, in this study, the CG did not fill out the motivation inventory because the wording of the inventory was primarily targeted toward the VR game system. In the future, the inventory should be modified to focus on the process of studying anatomy. By modifying it in this way, we could use it to compare the motivation among all 3 conditions.

## Conclusion

The current study developed a VR gaming system based on the HTC Vive environment. In this VR gaming environment, students learned anatomy knowledge, and the students who used the VR system had better retention of learning effects than those who did not. Our VR gaming system could be an excellent online learning tool. Students could master anatomy knowledge through this powerful and engaging learning method, while teachers could use our system for quizzes or assignments. Furthermore, introducing a multi-player competition had more positive effects on learning than playing the game as a single player. Developing competition between teams in the game is the next step in the development of our system.

## Data Availability

The datasets used and/or analyzed during the current study are available from the corresponding author on reasonable request.

## Abbreviations

VR: Virtual reality
CG: Control group
SP: Single-player
MP: Multiple-player
